# Zonisamide is effective in the preventive therapy of chronic migraine

**DOI:** 10.1186/1129-2377-1-S14-P215

**Published:** 2013-02-21

**Authors:** R Belvis, A Aceituno, M Martinez-Corral

**Affiliations:** 1 Dexeus University Insitute, Spain

## Introduction

Topiramate is the only approved oral preventive drug for chronic migraine (CM). However, we usually use other preventive drugs of prevention of frequent episodic migraine. Zonisamide is a neuromodulator that has showed efficacy in prevention of CM.

## Objectives

To evaluate the efficacy of zonisamide after three months of therapy in CM patients (IHS-2006 criteria) that present inefficacy/intolerance/contraindication to topiramate, sodium valproate, â-blocks and flunarizine.

## Methods

Doses ZNS: 50-200mg. Primary end-point: Number of migraine days-NMD: Ineffective response-Inef(reduction of days<50%), good-G(50-75%), excellent-Exc(>75%). Secondary end-points: number of migraine episodes-NME; number of headache days-NHD; drug overuse; consumption of triptans and NSAIDs; EVA and MIDAS score, effective mean dose of ZNS, adverse events.

## Results

We prospectively included 27 patients (May 2011-May 2012), 85.2% women. Twelve patients (44.4%) presented 16 adverse events (100% mild). Four of them-14.8% left the therapy. NMD, in the 23 patients who finished the three months follow-up, was Exc-39.1%, G-43.5%, and Inef-17.4%; NME was Exc-30.4%, G-43.5%, and Inef-26.1%; and NHD was Exc-34.8%, G-39.1%, and Inef-26.1%. Drug overuse disappeared in 47.8% of overusers. The consumption of triptans and NSAIDs decreased 54.3%, and 65.7%. The EVA score decreased 3.7 points (56.2%), and MIDAS scale score decreased 14.6 points (48.7%). Effective mean doses of ZNS: 106.6mg/night.

## Conclusion

Zonisamide presents a good profile of efficacy (70.3% of responses) and an acceptable tolerance. It could be a useful therapeutic option in CM patients intolerants, refractory, or with contraindications for topiramate, and the other drugs preventive drugs of migraine.
